# The N-linker region of hERG1a upregulates hERG1b potassium channels

**DOI:** 10.1016/j.jbc.2022.102233

**Published:** 2022-07-05

**Authors:** Ashley A. Johnson, Taylor R. Crawford, Matthew C. Trudeau

**Affiliations:** Department of Physiology, University of Maryland School of Medicine, Baltimore, Maryland, USA

**Keywords:** KCNH2, hERG1a, hERG1b, PAS domain, eag domain, N-linker domain, FRET, biochemical pull-down interaction assay, long QT syndrome, biotinylation, cDNA, complementary DNA, CFP, cyan fluorescent protein, CNBHD, cyclic nucleotide–binding homology domain, GST, glutathione-*S*-transferase, G–V, conductance–voltage, HEK293, human embryonic kidney 293 cell line, hERG1, human Ether-á-go-go-Related Gene, IKr, rectifier potassium current, LQTS, long QT syndrome, mCFP, monomeric CFP, O.N., overnight, PAS, Per–Arnt–Sim, TEVC, two-electrode voltage clamp

## Abstract

A major physiological role of hERG1 (human Ether-á-go-go-Related Gene 1) potassium channels is to repolarize cardiac action potentials. Two isoforms, hERG1a and hERG1b, associate to form the potassium current I_K__r_ in cardiomyocytes. Inherited mutations in hERG1a or hERG1b cause prolonged cardiac repolarization, long QT syndrome, and sudden death arrhythmia. hERG1a subunits assemble with and enhance the number of hERG1b subunits at the plasma membrane, but the mechanism for the increase in hERG1b by hERG1a is not well understood. Here, we report that the hERG1a N-terminal region expressed in *trans* with hERG1b markedly increased hERG1b currents and increased biotin-labeled hERG1b protein at the membrane surface. hERG1b channels with a deletion of the N-terminal 1b domain did not have a measurable increase in current or biotinylated protein when coexpressed with hERG1a N-terminal regions, indicating that the 1b domain was required for the increase in hERG1b. Using a biochemical pull-down interaction assay and a FRET hybridization experiment, we detected a direct interaction between the hERG1a N-terminal region and the hERG1b N-terminal region. Using engineered deletions and alanine mutagenesis, we identified a short span of amino acids at positions 216 to 220 within the hERG1a “N-linker” region that were necessary for the upregulation of hERG1b. We propose that direct structural interactions between the hERG1a N-linker region and the hERG1b 1b domain increase hERG1b at the plasma membrane. Mechanisms regulating hERG1a and hERG1b are likely critical for cardiac function, may be disrupted by long QT syndrome mutants, and serve as potential targets for therapeutics.

The human Ether-á-go-go-Related Gene (hERG, KCNH2) encodes a voltage-activated potassium channel that is critical in human health and disease. Two isoforms of the hERG gene, hERG1a and hERG1b, form the rapid component of the delayed rectifier potassium current (I_Kr_) in heart (1, 2, 3, 4, 5) which helps to repolarize the ventricular action potential (6). Mutations in hERG1a (7) or hERG1b (8) are linked to long QT syndrome (LQTS), a predisposition to prolonged action potentials, cardiac arrhythmia, and sudden death. hERG channels are the targets for acquired LQTS, which is due to the off-target inhibition of hERG channels by drugs (5) and is a common clinical problem (9).

hERG1a and hERG1b isoforms have different structural and functional properties that are due in part to their divergent N-terminal regions. The hERG1a N-terminal region has 398 amino acids, whereas the N-terminal region of hERG1b has 58 amino acids (Fig. 1, *A* and *B*). The N-terminal region of hERG1a contains a PAS (Per–Arnt–Sim) domain and PAS-CAP, which together encode the first 135 amino acids of hERG1a. The first 135 amino acids of hERG1a were initially termed the “eag domain” (10), but here, we will follow the recent suggestion that the first 135 amino acids be referred to as the PAS domain (Fig. 1*A*) for clarity (11). In hERG1a, the PAS domain is followed by an N-linker region, encoded by amino acids 136 to 398, which connects the PAS domain and the S1 transmembrane domain (Fig. 1*A*). The function of the N-linker is not well understood. In contrast to mammalian ERG1a, mammalian ERG1b does not contain a PAS domain or N-linker region and instead the first 36 amino acids of hERG1b are unique (3) and not conserved with other proteins (Fig. 1*B*). hERG1a and hERG1b are identical from N-terminal residues 377 in hERG1a and 37 in hERG1b through the carboxyl terminus (Fig. 1, *A* and *B*). hERG1a channels are characterized by a slow time course of deactivation (closing) that requires the PAS domain (10, 12, 13, 14) and the C-terminal CNBHD (cyclic nucleotide–binding homology domain) (12, 13). The mechanism for slow deactivation involves a direct structural interaction between the PAS and CNBHD in an intersubunit domain-swapped arrangement (12, 13) that is validated by a cryo-EM structure of the hERG1a channel (15). The PAS–CNBHD interaction is conserved in KCNH1 (EAG) channels (16) and may be a defining feature of KCNHs. Mouse and human ERG1b channels have rapid deactivation (3, 17) that is due to the lack a PAS domain (17). Slow deactivation can be introduced to hERG1b channels by coexpression in *trans* with hERG1a PAS domains (17), indicating that in hERG1a/hERG1b heteromeric channels, the hERG1b CNBHD likely makes an interaction with the PAS domain of hERG1a (17).

**Figure 1:**
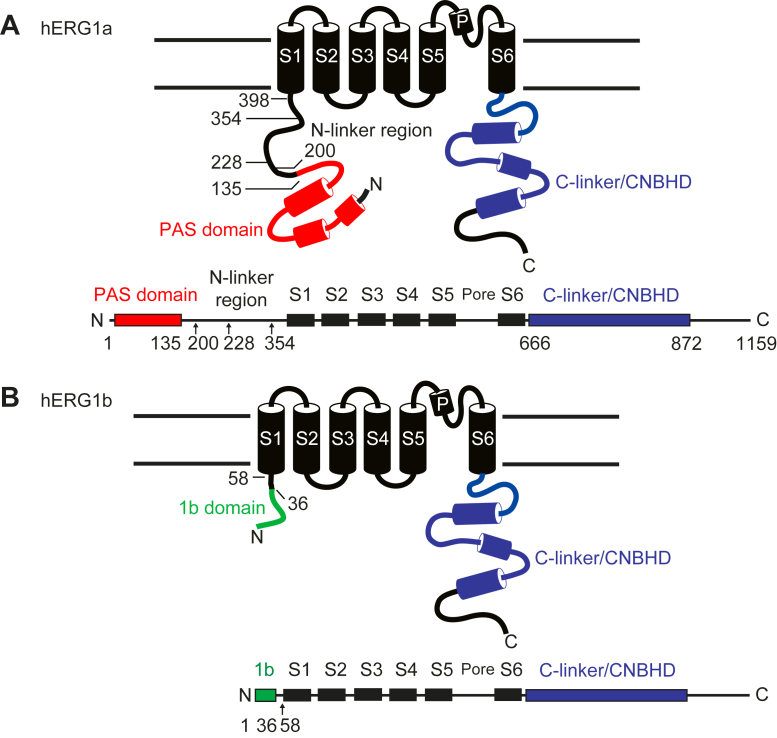
**Scheme of hERG1a and hERG1b subunits.** hERG1a and hERG1b contain six transmembrane domains and C-terminal C-linker and CNBHDs. hERG1a and hERG1b diverge in the N-terminal regions. *A*, the N-terminal region of hERG1a is comprised of 398 amino acids and contains a PAS domain and N-linker region. *B*, the hERG1b N-terminal region lacks a PAS domain and N-linker region and instead contains 58 amino acids, the first 36 of which are unique and referred to as the 1b domain. CNBHD, cyclic nucleotide–binding homology domain; hERG1, human Ether-á-go-go-Related Gene 1; PAS, Per–Arnt–Sim.

hERG1a subunits form homomeric channels with large currents in heterologous expression systems (4, 5, 18). In contrast, hERG1b (and mouse ERG1b) can form homomeric channels, but the currents are very small, as hERG1b is preferentially retained at intracellular membranes due in part to a diarginine retention signal in its unique N-terminal domain (3, 17, 19, 20). Mammalian ERG1a and ERG1b subunits directly associate in heterologous and native cells to form heterotetrameric channels (1, 2, 3, 8, 19, 20, 21, 22, 23). Coexpression of hERG1a with hERG1b increases the amount of “mature” (*i.e.*, N-linked glycosylated) hERG1b on a Western blot (20), suggesting that hERG1a increases hERG1b destined for the plasma membrane. A biochemical interaction was detected between fusion proteins encoding the N-terminal region–S2 transmembrane domain of hERG1a and N-terminal region–S2 of hERG1b (19), but the functional correlate of this interaction is not clear, and more precise molecular determinants for the N-terminal S2 interaction have not been identified.

In this study, we used hERG1a N-terminal regions (composed of the PAS domain and N-linker and bearing deletions or mutations) encoded by mRNAs in *Xenopus* oocytes or encoded by complementary DNAs (cDNAs) in human embryonic kidney 293 (HEK293) cells and coexpressed in *trans* with hERG1b channels to simultaneously probe functional and structural interactions. Using hERG1a N-terminal regions expressed in *trans* with hERG1b channels has several advantages as an experimental approach: (1) changes in hERG1b channel properties can be attributed directly to the hERG1a N-terminal regions, providing a direct link between structure of the hERG1a N-terminal region and hERG1b channel function, (2) changes in hERG1b channel properties are not obscured by the ion-conducting properties of intact hERG1a channels, as in previous studies, and (3) since the hERG1a PAS domain regulates hERG1b channel–gating properties, regulated gating is an independent internal positive control for expression of hERG1a N-terminal regions.

Here, we report that the hERG1a N-terminal region encoded by amino acids 1 to 354 (hERG1a N1–354), which is composed of the PAS domain and most of the N-linker (Fig. 1*A*), increased hERG1b, as measured by an increase in hERG1b ionic current recorded with two-electrode voltage clamp (TEVC) or whole-cell patch clamp and by an increase in the amount of “mature” hERG1b at the plasma membrane as measured with surface biotinylation and Western blot assays. hERG1b channels with a deletion of the unique N-terminal 1b domain did not have a measurable increase in current or biotinylated protein when coexpressed with hERG1a N1–354, indicating that the 1b domain was required for the increase in hERG1b. Using biochemical pull-down interaction assays and FRET two-hybrid interaction assays, we detected an interaction between the hERG1a N-terminal region and the hERG1b N-terminal region. The hERG1a N-terminal region–dependent increase of hERG1b required the N-linker, since a hERG1a N-terminal region encoding just the PAS domain (N1–135) expressed in *trans* with hERG1b did not measurably increase hERG1b currents or biotinylated hERG1b protein. Using hERG1a N-terminal regions with sequential deletions within the N-linker, we determined that residues 200 to 228 of the N-linker were required for the increase in hERG1b. We used alanine mutagenesis to further examine the N-linker region bounded by amino acids 200 and 228 and determined that residues 216 to 220 in the hERG1a N-linker region were necessary for the increase in hERG1b. Mechanisms that enhance hERG1b current are anticipated to shorten action potentials, which could be antiarrhythmic, and may point toward hERG1b or the hERG1a N-linker region as molecular targets for therapy for LQTS.

## Results

**Figure 2:**
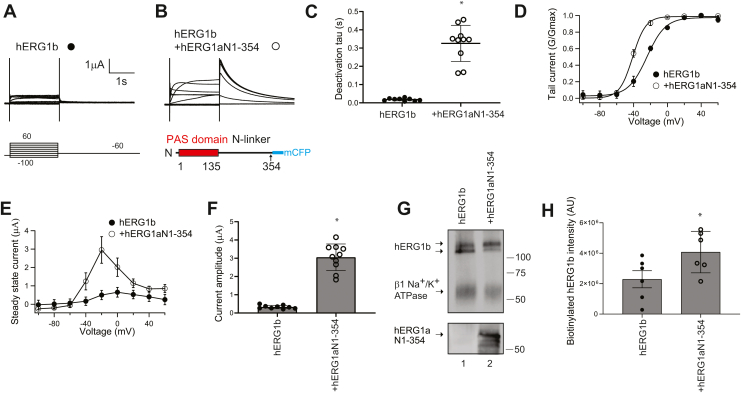
**hERG1a N-terminal region (hERG1a N1–354) increased hERG1b currents and surface expression.** Two-electrode voltage-clamp recordings of (*A*) hERG1b (*closed circles*) and (*B*) hERG1a N1–354 (as depicted in the scheme) coexpressed in *trans* with hERG1b (*open circles*). Voltage pulse protocol as indicated. The scale bar represents 1 μA and 1 s. *C*, time constant of deactivation measured at −60 mV. *D*, conductance–voltage (G–V) relationship. The *solid line* is a Boltzmann fit to the data. *E*, current–voltage (I–V) relationship. *F*, peak current measured at a depolarizing voltage command from data in *A* and *B*. *G*, Western blot of surface biotinylated hERG1b expressed individually (lane 1) and hERG1a N1–354 in *trans* with hERG1b (lane 2) blotted with the anti-hERG KA antibody to detect hERG1b. We used an antibody to detect the beta subunit of the endogenous Na/K-ATPase as a positive loading control. An anti-GFP antibody that detected the mCFP epitope of hERG1aN1–354 was used as an input control in a parallel blot. *H*, plot of densitometry of data from *G*. N ≥ 3 for each. Error bars represent mean ± SD. ∗ denotes *p* ≤ 0.05 by ANOVA. CFP, cyan fluorescent protein; mCFP, monomeric CFP; hERG1, human Ether-á-go-go-Related Gene 1.

To carry out these experiments, we expressed hERG1b fused to Citrine fluorescent protein (here termed hERG1b for clarity, see the Experimental procedures section) in *Xenopus* oocytes and used TEVC to record typical hERG1b currents (Fig. 2*A*). We next coexpressed an hERG1a N-terminal region, composed of the PAS domain and most of the N-linker region fused to cyan fluorescent protein (CFP) (here termed hERG1a N1–354 for clarity) in *trans* with hERG1b (Fig. 2*B*). Compared with hERG1b channels, which were characterized by rapid channel deactivation, we found that hERG1a N1–354 regulated and slowed the deactivation time course of hERG1b (Fig. 2*C* and Table 1) and shifted the steady-state activation relationship of hERG1b toward more negative voltages (Fig. 2*D* and Table 1). We also report here that, in contrast to the relatively small ionic currents characteristic of hERG1b channels, that hERG1a N1–354 markedly increased the magnitude of hERG1b currents (Fig. 2, *E* and *F*). To directly test whether the hERG1a N1–354 increased the number of hERG1b channels at the plasma membrane, we measured surface biotinylated hERG1b using SDS-PAGE and Western blot analysis in HEK293 cells (Fig. 2*G*). Biotinylated hERG1b was detected as an upper band at 120 kDa and a lower band at 110 kDa on a Western blot, as labeled (Fig. 2*G*, lane 1). Mature (*upper*) hERG1b bands were faint as measured with densitometry (Fig. 2*H*), consistent with small hERG1b currents (Fig. 2, *A*, *E* and *F*). In marked contrast, hERG1a N1–354 coexpressed in *trans* with hERG1b increased the intensity of the mature hERG1b band (Fig. 2*G*, lane 2; *H*). Our results suggest that the hERG1a N-terminal region (hERG1a N1–354) increased hERG1b currents by increasing the number of hERG1b channels at the plasma membrane.

**Table 1:** Properties of hERG channels expressed in *Xenopus* oocytes

Channel	Current amplitude (μA)	SD	Time constant of deactivation (ms)	SD	V_1/2_	κ
hERG1b	0.67	0.19	0.03	0.01	−24.1 ± 1.19	10.944 ± 1.07
hERG1b + hERG1aN1–135	0.86	0.04	0.37	0.05	−32.639 ± 0.76	9.3495 ± 0.63
hERG1b + hERG1aN1–200	0.87	0.24	0.34	0.20	−36.643 ± 0.59	7.7255 ± 0.59
hERG1b + hERG1aN1–228	2.42	0.99	0.40	0.09	−43.644 ± 0.85	8.537 ± 0.80
hERG1b + hERG1aN1–354	2.27	0.84	0.35	0.03	−41.165 ± 0.51	7.7639 ± 0.56
hERG1bΔ2–36	0.45	0.02	0.03	0.01	−25.586 ± 4.15	11.823 ± 3.68
hERG1bΔ2–36 + hERG1aN1–354	0.85	0.21	0.29	0.06	−57.73 ± 0.661	7.9077 ± 0.69
hERG1b + hERG1aN1–228 (TPAAP→AAAAA)	2.25	0.38	0.33	0.04	−43.844 ± 0.15	7.4301 ± 0.14
hERG1b + hERG1aN1–228 (SSESL→AAAAA)	1.20	0.12	0.34	0.06	−46.403 ± 0.51	7.6938 ± 0.40
hERG1b + hERG1aN1–228 (ALDEV→AAAAA)	3.56	1.11	0.29	0.13	−44.762 ± 0.91	7.7667 ± 0.81
hERG1b + hERG1aN1–228 (TAMDN→AAAAA)	0.73	0.31	0.44	0.08	−41.812 ± 0.31	7.1849 ± 0.36
hERG1b + hERG1aN1–228 (HVAGL→AAAAA)	2.05	0.72	0.48	0.05	−42.842 ± 0.72	6.4448 ± 0.83

**Figure 3:**
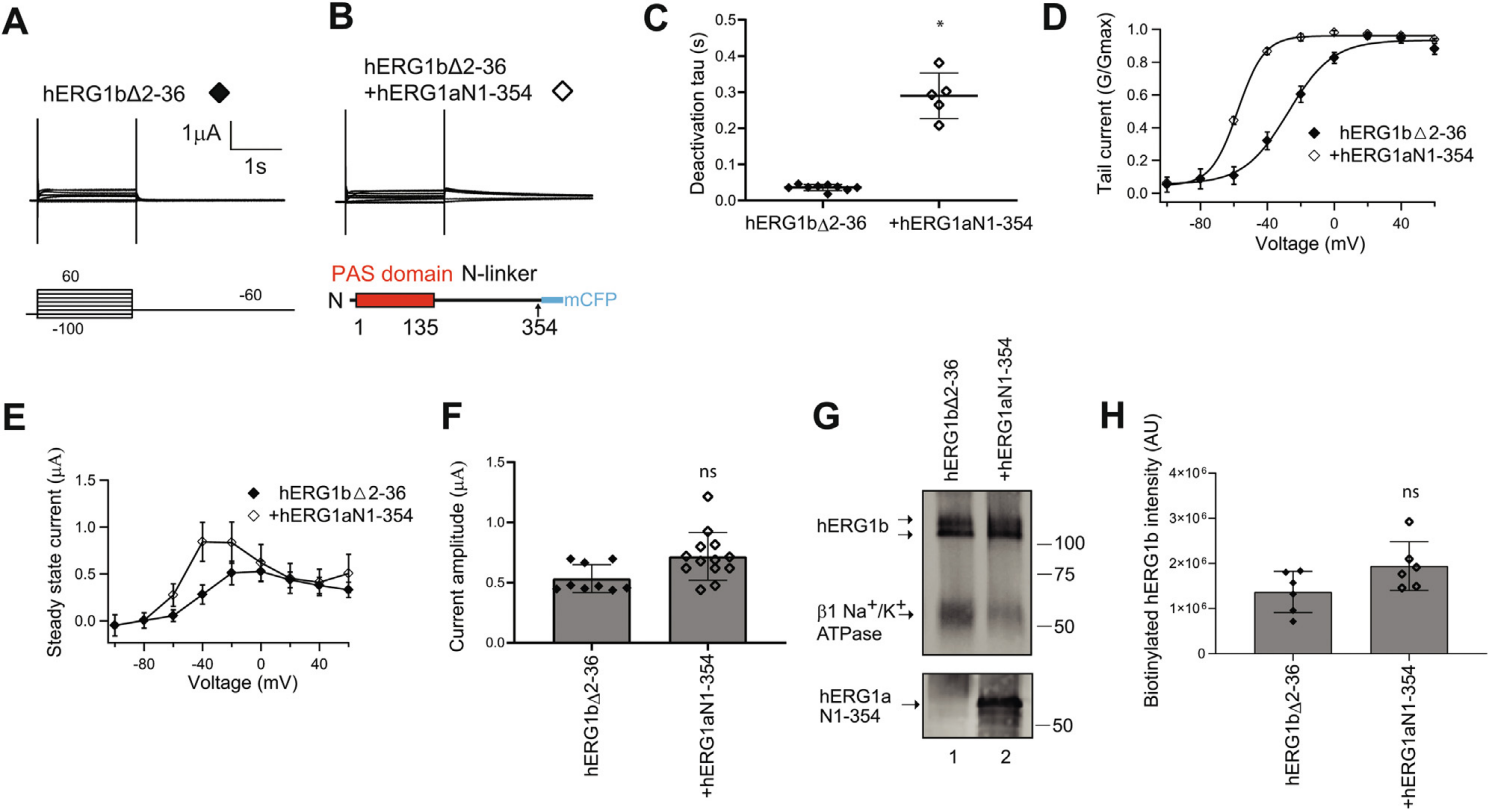
**hERG1a N1–354 does not measurably increase currents or surface expression of hERG1b channels in which the 1b domain is deleted (hERG1b Δ2–36).** Two-electrode voltage-clamp recordings of (*A*) hERG1b Δ2–36 (*closed diamonds*) and (*B*) hERG1aN-354 coexpressed in *trans* with hERG1b Δ2–36 (*open diamonds*). Voltage pulse protocol as indicated. The scale bar represents 1 μA and 1 s. *C*, plot of time constant of deactivation measured at −60 mV. *D*, conductance–voltage (G–V) relationship. The *solid line* is a Boltzmann fit to the data. *E*, current–voltage (I–V) relationship. *F*, peak current measured at a depolarizing voltage command from data in *A*. *G*, Western blot of surface biotinylated hERG1b Δ2–36 expressed individually (lane 1) and hERG1aN1–354 in *trans* with hERG1b Δ2–36 (lane 2) blotted with the anti-hERG KA antibody. The beta subunit of the Na/K-ATPase served as a positive loading control, and an anti-GFP antibody detected hERG1aN1–354 as an input control in a parallel blot. *H*, plot of densitometry of data from *G*. N ≥ 3 for each. Error bars represent mean ± SD. ∗ denotes *p* ≤ 0.05 by ANOVA. ns denotes not significantly different. hERG1, human Ether-á-go-go-Related Gene 1.

To identify functional determinants in hERG1b that were necessary for the increase in hERG1b by hERG1a N1–354, we generated a hERG1b channel that lacked the unique hERG1b N-terminal 1b domain fused to Citrine fluorescent protein (hERG1b Δ2–36) and recorded currents from oocytes expressing hERG1bΔ2–36 channels (Fig. 3*A*) and hERG1a N1–354 coexpressed in *trans* with hERG1bΔ2–36 channels (Fig. 3*B*). Like hERG1b channels, hERG1bΔ2–36 channels had currents with rapid deactivation (Fig. 3*C*), a conductance–voltage (G–V) plot midpoint of −25 ± 4 mV (Fig. 3*D* and Table 1), small outward currents (Fig. 3, *E* and *F*), and a low density of “mature” protein at the plasma membrane (Fig. 3*G*, lane 1; Fig. 3*H*), which were all properties similar to that of wildtype hERG1b channels (compare with Fig. 2*A*, *C*–*H* and Table 1). When hERG1a N1–354 was coexpressed in *trans* with hERG1bΔ2–36, we measured slower deactivation (Fig. 3*C*) and a leftward shift in the conductance–activation curve (Fig. 3*D*), which were comparable to hERG1a N1–354 regulation of wildtype hERG1b (Fig. 2, *A*–*D*). In contrast, we did not detect a measurable increase in ionic currents (Fig. 3, *E* and *F*) or biotinylated protein (Fig. 3*G* lane 2, Fig. 3*H*). These results show that the hERG1b N-terminal 1b domain was necessary for hERG1b to be increased by hERG1a N1–354.

We detected a larger leftward shift in the voltage dependence of activation of hERG1a N1–354 coexpressed in *trans* with hERG1b Δ2–36 than in hERG1a N1–354 coexpressed in *trans* with hERG1b (Figs. 2*D* and 3*D* and Table 1). This finding was unexpected as the 1b domain had previously been associated with housing the RXR trafficking motif but not as a determinant of gating *per se*. The mechanism for this leftward shift is not clear, but it is consistent with an additional role of the 1b domain in hindering activation of hERG1b by hERG1a N1–354. Further experiments will be required to make this determination.

**Figure 4:**
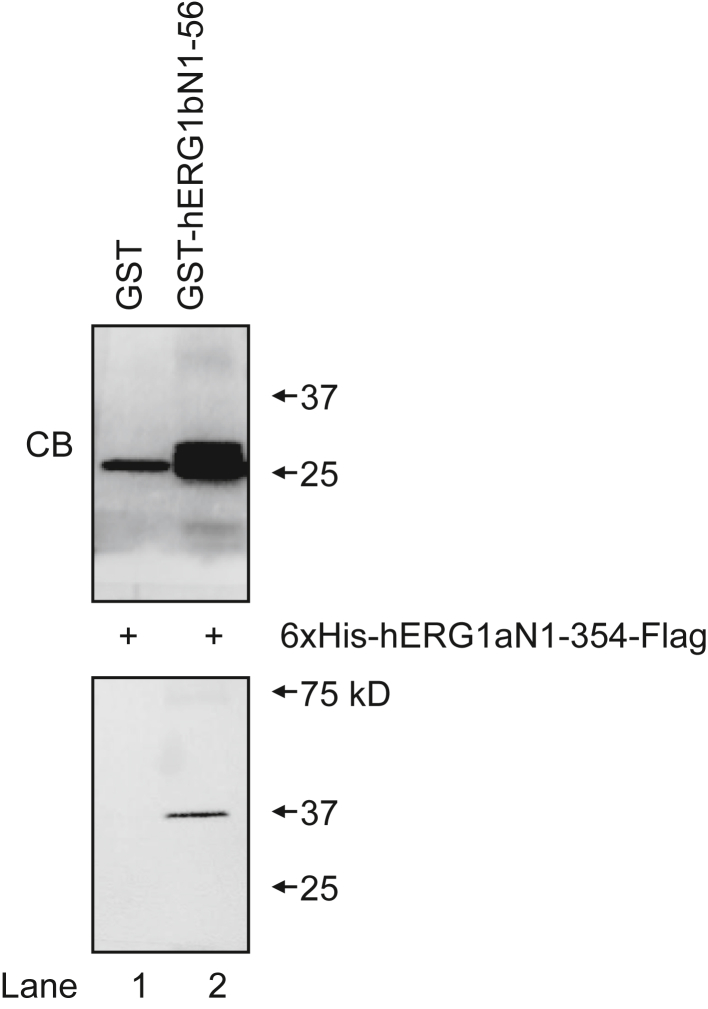
**The hERG1b N-terminal region (hERG1b N1–56) interacts with the hERG 1a N-terminal region (hERG1a N1–354) in a biochemical pull-down interaction assay**. GST or GST–hERG1b N-terminal region was incubated with hERG1a N1–354 as indicated (+). *Upper panel*, Coomassie blue (CB)–stained gel of GST and GST-hERG1b N1–56 inputs. *Lower panel*, Western blot following GST (lane 1) or GST-hERG1b N1–56 (lane 2) incubation with hERG1a N1–354. The band at 37kD in lane 2 corresponds to the hERG1a N1–354 and indicates a specific interaction with hERG1b N1–56. hERG1a N1–354 proteins were detected with an anti-FLAG antibody. N = 3. GST, glutathione-*S*-transferase; hERG1, human Ether-á-go-go-Related Gene 1.

As the hERG1b N-terminal 1b domain was required for hERG1b upregulation by hERG1a N1–354, we next performed biochemical pull-down interaction assays to test for a direct interaction between the hERG1b N-terminal region and hERG1a N1–354 (Fig. 4). An advantage of the pull-down technique is that the two fusion proteins that interact in this assay are considered to be sufficient to make a direct protein–protein interaction. We generated the N-terminal region of hERG1b as a glutathione-*S*-transferase (GST) fusion protein (GST-hERG1b N1–56) and hERG1a N-terminal region as a 6×-histidine fusion protein with a FLAG epitope for detection (6xHis-hERG1a N1–354-FLAG). We expressed each fusion protein in bacteria and purified them using glutathione beads or nickel columns, respectively. GST and GST-hERG1b N1–56 inputs are shown on the Coomassie blue–stained gel (Fig. 4, *upper panel*). We incubated GST or GST-hERG1b N1–56 with 6xHis-hERG1a N1–354-FLAG, purified putative protein complexes using glutathione beads, and performed SDS-PAGE and Western blot analysis. We did not detect 6xHis-hERG1a N1–354-FLAG in negative control experiments with GST (Fig. 4, *lower panel*, lane 1). In contrast, we detected a band corresponding to the 6xHis-hERG1a N1–354-FLAG at its predicted molecular weight (37 kD; Fig. 4, *lower panel*, lane 2) indicating that hERG1a N1–354 made an interaction with hERG1bN1–56, meaning that the hERG1a and hERG1b N-terminal regions interacted.

**Figure 5:**
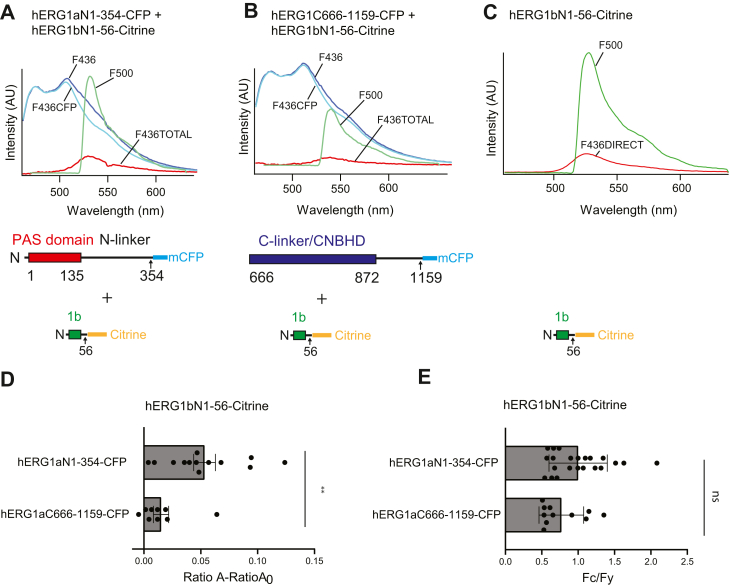
**The hERG1b N-terminal region (hERG1b N1–56) and the hERG1a N-terminal region (hERG1a N1–354) associate in a FRET two-hybrid assay.***A*, plot of fluorescence intensity (A.U.) *versus* wavelength (nanometer) from cells expressing hERG1a N1–354-mCFP + the hERG1b N1–56-Citrine where F_436_ (*blue trace*) is the emission spectrum measured with a FRET cube and F_500_ (*green trace*) is the emission spectrum measured with a YFP cube. F_436CFP_ (*cyan trace*) is a scaled emission spectrum of a control cell expressing hERG1a N1–354-mCFP measured with a FRET cube, F_436total_ is F_436_ − F_436CFP_. *B*, plot of the hERG1 C666–1159-mCFP and hERG1b N1–56-Citrine where F_436_ (*blue trace*) is the emission spectrum measured with a FRET cube and F_500_ (*green trace*) is the emission spectrum measured with a YFP cube. F_436CFP_ (*cyan trace*) is a scaled emission spectrum of a control cell expressing hERG1a 1–354-mCFP measured with a FRET cube, F_436total_ is F_436_ − F_436CFP_. *C*, plot of fluorescence intensity *versus* wavelength from a control cell expressing hERG1b N1–56-Citrine, where F_436direct_ is the emission spectrum measured with a FRET cube and F_500_ is the emission spectrum measured with a YFP cube. *D*, plot of Ratio A − Ratio A_0_ (a value proportional to FRET) for each pair of hERG proteins, as indicated. *E*, plot of peak CFP fluorescence intensity (Fc) divided by peak Citrine fluorescence intensity (Fy), as a control for similar amounts of input. N ≥ 3 for each. Error bars represent mean ± SD. ∗∗ denotes *p* ≤ 0.01 by ANOVA. CFP, cyan fluorescent protein; hERG1, human Ether-á-go-go-Related Gene 1; mCFP, monomeric CFP; ns, not significantly different.

In an experiment which complements the biochemical pull-down interaction assay, we next examined interactions between the entire hERG1b N-terminal region and the hERG1a intracellular N- and C-terminal regions using a FRET two-hybrid assay (Fig. 5). In a FRET two-hybrid experiment (24), two protein regions of interest are each fused to different fluorescent proteins that are FRET pairs (we used mCFP as the FRET donor and Citrine as the FRET acceptor) as in previous studies (12). The cDNAs encoding two protein regions fused to FRET pairs are cotransfected, and the regions are allowed to freely associate in the milieu of a biological cuvette (*e.g.*, a HEK293 cell). If FRET is detected in this experiment, it suggests that the two protein regions associated and brought the mCFP and Citrine within sufficient proximity (within approximately 80 Å) for energy transfer (12, 24).

For this assay, we generated the hERG1b N-terminal region fused to Citrine fluorescent protein (hERG1b N1–56-Citrine) and used the hERG1a N-terminal region fused to mCFP (hERG1a N1–354-mCFP) as well as the hERG1 C-terminal region fused to mCFP (hERG1 C666–1159-mCFP) (Fig. 5, *A* and *B*) from a previous study (12, 24). The C-terminal region is identical in hERG1a and hERG1b (Fig. 1).

As in previous work, we used spectral separation and ratiometric analysis to measure FRET (12, 24). Advantages of this approach are that it corrects for direct excitation of the acceptor by light at wavelengths intended to excite the donor (crosstalk), overlap between the donor and acceptor emission spectra (bleed-through), and cell-to-cell variations in the intensity of the donor or acceptor because of differences in expression levels.

To perform FRET spectroscopy from single cells coexpressing either hERG1a N1–354-mCFP + hERG1b N1–56-Citrine (Fig. 5*A*) or hERG1 C666–1159-mCFP + hERG1b N1–56-Citrine (Fig. 5*B*), we measured emission spectra with a FRET cube (F_436_, *blue trace*, Fig. 5, *A* and *B*) and with a YFP cube (F_500_, *green trace*, Fig. 5, *A* and *B*). To correct for “bleed-through,” a CFP spectrum (F_436CFP_, *cyan trace*, Fig. 5, *A* and *B*) was measured in separate control experiments from cells expressing hERG1a N1–354-mCFP, scaled, and subtracted from each F_436_ spectra to yield the “total spectra” (F_436total_, *red trace*, Fig. 5, *A* and *B*). The ratio of F_436total_ to F_500_ is defined as Ratio A

(1) $$\text{Ratio}\text{A}=\frac{{\text{F}}_{436\text{total}}}{{\text{F}}_{500}}=\left(\frac{{\text{F}}_{436\text{direct}}}{{\text{F}}_{500}}\right)+\left(\frac{{\text{F}}_{436\text{FRET}}}{{\text{F}}_{500}}\right)$$

and is calculated to control for cell-to-cell variation in expression levels and contains a component due to direct emission of Citrine by 436 nm light (F_436direct_/F_500_) and a component proportional to FRET efficiency (F_436FRET_/F_500_).

To determine the F_436FRET_/F_500_ component, we measured the emission spectra with a FRET cube (F_436direct_, *red trace*, Fig. 5*C*) and with a YFP cube (F_500_, *green trace*, Fig. 5*C*) in a separate control experiment from cells expressing hERG1b N1–56-Citrine from which the ratio of the spectra (F_436direct_/F_500_) is defined as Ratio A_0_

(2) $$\text{Ratio }A_{0}=\left(\frac{{\text{F}}_{436\text{direct}}}{{\text{F}}_{500}}\right)$$

and is a control measurement of “crosstalk” in the imaging system.

Next, we subtracted Ratio A_0_ (Equation 2) from Ratio A (Equation 1),

(3) $$\text{Ratio}\text{A}-\text{Ratio }A_{0}=\left(\frac{{\text{F}}_{436\text{FRET}}}{{\text{F}}_{500}}\right)$$

which yielded F_FRET436_/F_500_, which is proportional to FRET efficiency (25, 26) and is reported here (Fig. 5*D*). A Ratio A—Ratio A_0_ value greater than zero indicates FRET, and we detected robust FRET between hERG1b N1–56-Citrine and hERG1a N1–354-mCFP (Fig. 5, *A* and *D*). These results suggest that the hERG1a N-terminal region makes an interaction with the hERG1b N-terminal region and directly supports our similar results and conclusion from the biochemical pull-down interactions (Fig. 4) and electrophysiology and biotinylation experiments (Figs. 2 and 3). We did not detect measurable FRET between the hERG1b N-terminal region (hERG1b N1–56-Citrine) and the hERG1 C-terminal region (hERG1 C666–1159-mCFP) (Fig. 5. *B* and *D*). These results show that the interaction of the hERG1b N-terminal region with the hERG1a N-terminal region was specific.

As a final control for FRET donor input, we compared the relative peak fluorescence intensities from each of the two FRET donors compared with the FRET acceptor (F_C_/F_Y_) and found that the donor-to-acceptor ratio was similar (Fig. 5*E*). This meant that the absence of measurable FRET in Figure 5*B* was not simply because of insufficient donor levels.

**Figure 6:**
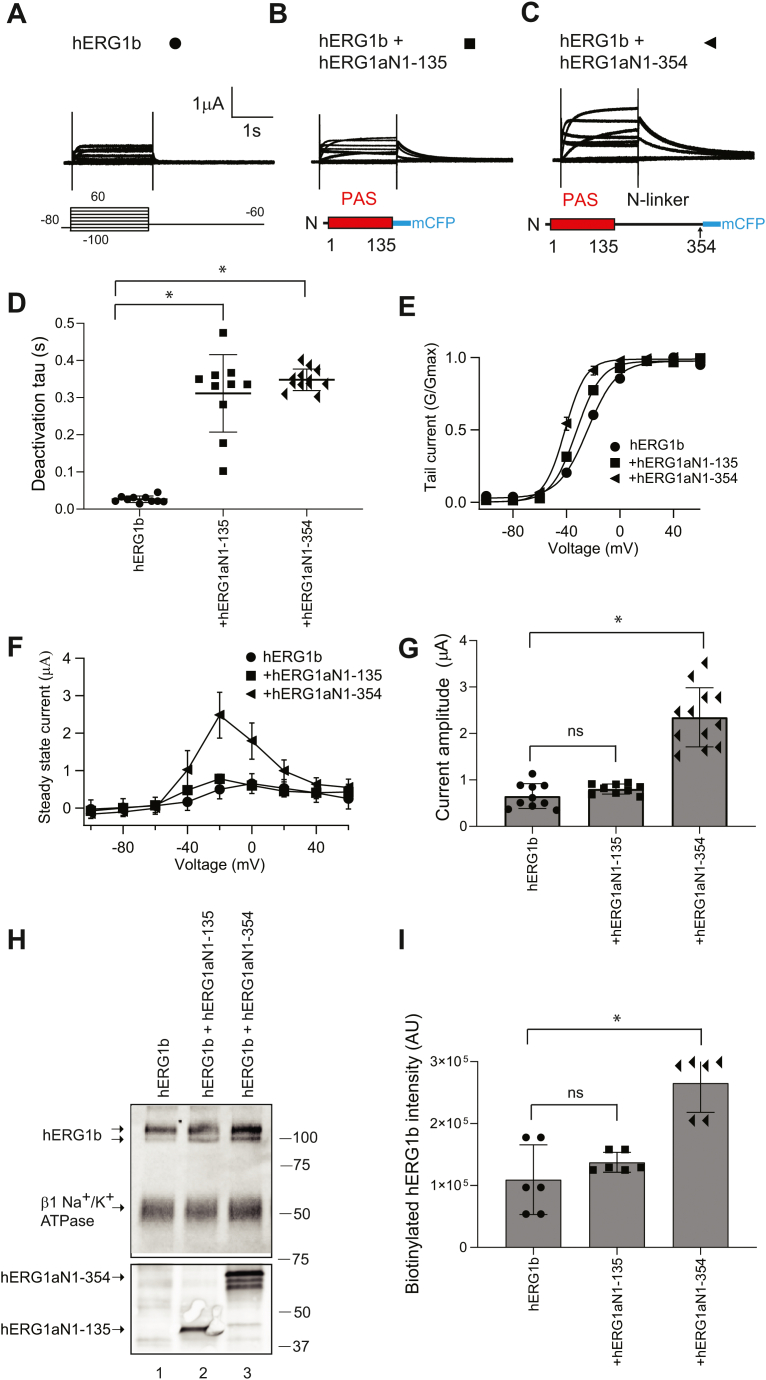
**The hERG1a PAS domain (hERG1a N1–135) did not increase hERG1b current.***A*, two-electrode voltage-clamp recordings of (*A*) hERG1b (*black circles*), (*B*) hERG1a N1–135 expressed in *trans* with hERG1b (*black squares*), and (*C*) hERG1a N1–354 expressed in *trans* with hERG1b (*black triangles*). Voltage protocol is indicated and is the same as for Figure 2. The scale bar represents 1 μA and 1 s. *D*, plot of time constant of deactivation at −60 mV. *E*, conductance–voltage (G–V) plot. *F*, current–voltage (I–V) plot. *G*, histogram of peak current amplitude at a depolarizing voltage from data in *A*–*C*. N ≥ 9 for each. Error bars represent mean ± SD. *F*, Western blot of surface biotinylated hERG1b coexpressed with control vector (lane 1), hERG1a N1–135 coexpressed in *trans* with hERG1b (lane 2), and hERG1a N1–354 coexpressed in *trans* with hERG1b (lane 3) where hERG1b was detected using the anti-hERG KA antibody. We used an antibody that detected the beta subunit of the Na^+^/K^+^ ATPase as a positive loading control. We used an anti-GFP antibody to detect hERG1a N1–135 and N1–354 proteins as input controls in a parallel blot. *G*, histogram of band intensity of biotinylated hERG1b. N ≥ 3 for each. Error bars represent mean ± SD. ∗ denotes *p* ≤ 0.05 by ANOVA. hERG1, human Ether-á-go-go-Related Gene 1; ns, not significantly different; PAS, Per–Arnt–Sim.

To identify functional and structural determinants within the hERG1a N-terminal region that were required for the increase in hERG1b, in side-by-side experiments, we recorded hERG1b (Fig. 6*A*), the hERG1a PAS domain (hERG1a N1–135) in *trans* with hERG1b (Fig. 6*B*), and hERG1a N1–354 in *trans* with hERG1b (Fig. 6*C*).

Compared with hERG1b, hERG1a N1–135 expressed in *trans* with hERG1b had a slower time course of deactivation, as anticipated (17), that was similar to that of hERG1a N1–354 when expressed in *trans* with hERG1b (Fig. 6*D* and Table 1). Compared with hERG1b, hERG1a N1–135 in *trans* with hERG1b had a slightly left-shifted G–V relationship, as anticipated (17), but not as left-shifted as hERG1a N1–354 in *trans* with hERG1b (Fig. 6*E* and Table 1). However, compared with hERG1b, there was not a measurable increase in magnitude of outward current for hERG1a N1–135 in *trans* with hERG1b (Fig. 6, *F* and *G*). In contrast, the currents for hERG1a N1–354 in *trans* with hERG1b were sixfold larger (Figs. 6, *F* and *G* and 2, *E* and *F*). Similar to experiments in oocytes, we found that whole-cell patch-clamp recordings from HEK293 cells were comparable to hERG1b (Fig. S1*A*). hERG1a N1–135 in *trans* with hERG1b (Fig. S1*B*) had slower deactivation gating (Fig. S1*C*) and a similar G–V relationship (Fig. S1*D*) but did not measurably increase the magnitude of outward hERG1b currents (Fig. S1, *E* and *F*). When hERG1a N1–135 was expressed in *trans* with hERG1b, we did not detect a measurable change in density of biotinylated hERG1b bands on a Western blot (Fig. 6*H*, lane 2; *I*), consistent with the lack of upregulation of hERG1b by the hERG1a N1–135 in electrophysiology experiments (Figs. 6, *A*, *F* and *G* and S1). In a side-by-side control experiment, hERG1a N1–354 increased “mature” hERG1b (Fig. 6*H*, lane 3; *I*). Together, our results confirm that hERG1a N1–135 (encoding the PAS domain) regulates gating in hERG1b, as previously reported (17, 24), but hERG1a N1–135 does not increase hERG1b channels at the plasma membrane. Instead, our results suggested that a region located between amino acids 136 and 354, encoding part of the hERG1a N-linker, was necessary for the increase in the magnitude of hERG1b currents and the amount of hERG1b channels at the plasma membrane.

**Figure 7:**
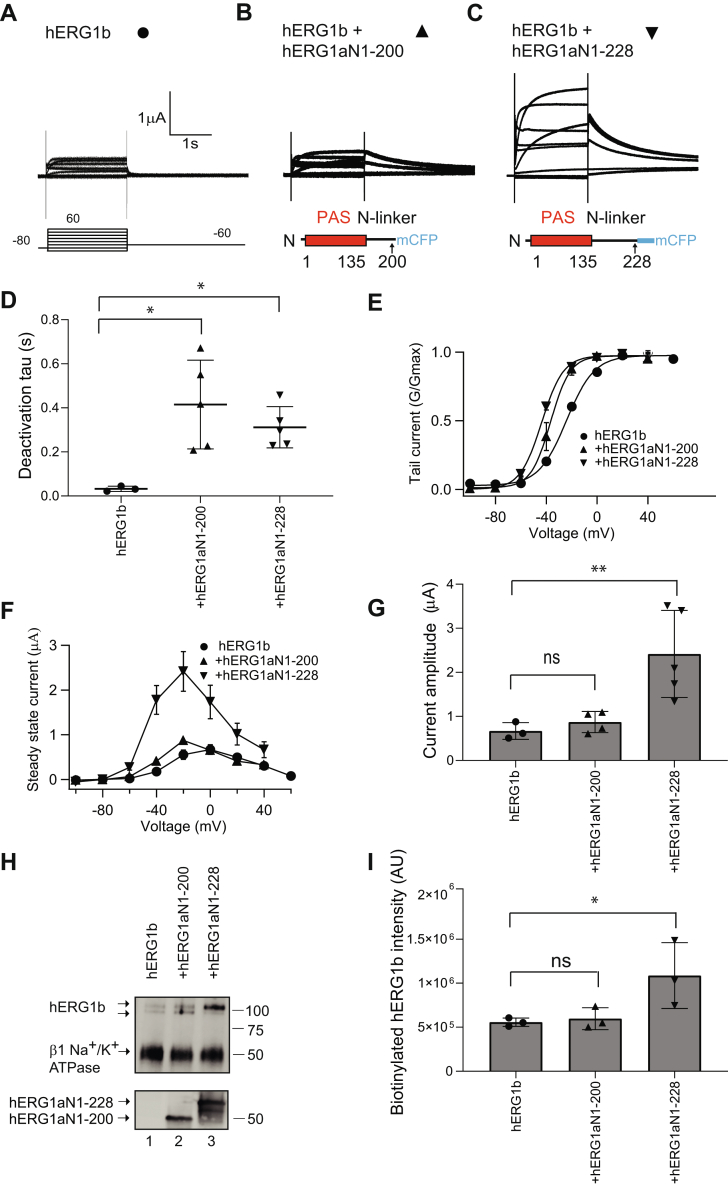
**hERG1a N-terminal regions composed of amino acids 1–228, but not 1–200, increased hERG1b current.***A*, two-electrode voltage-clamp recordings of (*A*) hERG1b (*closed circles*) and (*B*) hERG1a N1–200 coexpressed in *trans* with hERG1b (*closed upright triangle*), or (*C*) hERG1a N1–228 coexpressed in *trans* with hERG1b (*downward triangle*), as indicated. Voltage protocol is the same as for Figure 2. The scale bars represent 1 μA and 1 s. *D*, plot of time constant of deactivation at −60 mV. *E*, conductance–voltage (G–V) plot. *F*, current–voltage (I–V) plot. *G*, histogram of peak current amplitude at a depolarizing voltage from data in *A*. *H*, Western blot of surface biotinylated hERG1b coexpressed with control vector (lane 1), hERG1a N1–200 (lane 2), or hERG1a 1 to 228 (lane 3), and detected with anti-hERG KA. The Na^+^/K^+^ ATPase beta subunit was detected with an antibody as a loading control. An anti-GFP antibody detected hERG1a 1–220 or 1–228 in blots of input controls in a parallel blot. *I*, plot of densitometry (A.U.) of hERG1b from data as in *H*. N ≥ 3 for each. Error bars represent mean ± SD. ∗ denotes *p* ≤ 0.05 by ANOVA, and ∗∗ denotes *p* ≤ 0.01. hERG1, human Ether-á-go-go-Related Gene 1; ns, not significantly different.

To identify a region between hERG1a residues 136 and 354 that was required to increase hERG1b, we generated constructs that encoded the first 200 amino acids of the hERG1a N-terminal region (hERG1a N1–200) and another that encoded the first 228 amino acids of the hERG1a N-terminal region (hERG1a N1–228) (Fig. 7). Compared with hERG1b (Fig. 7*A*), we found that hERG1a N1–200 in *trans* with hERG1b (Fig. 7*B*) and hERG1a N1–228 in *trans* with hERG1b (Fig. 7*C*) each regulated and slowed hERG1b deactivation gating and each left shifted the hERG1b G–V relationship (Fig. 7, *D* and *E*). However, we found that hERG1a N1–200 did not enhance hERG1b outward currents or biotinylated hERG1b (Fig. 7,*F*–*I*), similar to hERG1a N1–135 (Fig. 6), whereas hERG1a N1–228 increased hERG1b currents and biotinylated hERG1b at the membrane (Fig. 7,*F*–*I*) similar to hERG1a N1–354 (Fig. 2). Similar to experiments in oocytes, whole-cell patch-clamp recordings from HEK293 cells showed that, compared with hERG1b (Fig. S2*A*), hERG1a N1–228 in *trans* with hERG1b (Fig. S2*B*) exhibited a slowed deactivation time course (Fig. S2*C*), little measurable effect on the G–V relationship (Fig. S2*D*), and markedly increased outward hERG1b currents (Fig. S2, *E* and *F*). The results here suggested that that the hERG1a PAS domain was insufficient to increase hERG1b currents and instead the region located between amino acids 200 and 228 in the hERG1a N-linker was necessary for the increase in hERG1b channels.

**Figure 8:**
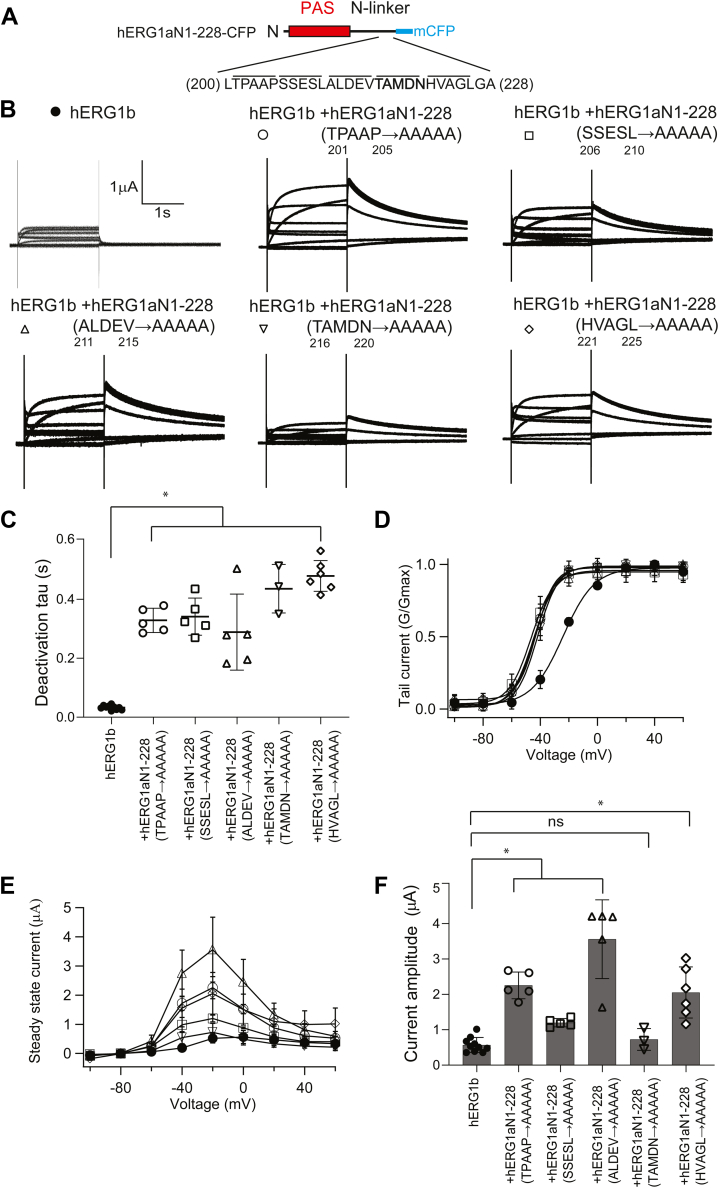
**Alanine mutagenesis revealed amino acids 216–220 of the hERG1a N-linker were necessary for increased hERG1b current amplitude.***A*, scheme depicting a hERG1a N1–228 with expanded view of amino acid sequence bounded by residues 200–228 with each line denoting a group of five sequential residues that were mutated to alanine. *B*, exemplar two-electrode voltage-clamp recordings of hERG1b and each of the five hERG1a N1–228s containing the alanine mutants ^201^TPAAP^205^ to ^201^AAAAA^205^, ^206^SSESL^210^ to ^206^AAAAA^210^, ^211^ALDEV^215^ to ^211^AAAAA^215^, ^216^TAMDN^220^, and ^221^HVAGL^226^ as labeled and individually coexpressed in *trans* with hERG1b. *C*, plot of time constant of deactivation at −60 mV. *D*, conductance–voltage (G–V) plot. *E*, current–voltage (I–V) plot. *F*, histogram of peak current amplitude at depolarizing voltage from data in *B*. N ≥ 3 for each. Error bars represent mean ± SD. ∗ denotes *p* ≤ 0.05 by ANOVA. hERG1, human Ether-á-go-go-Related Gene 1; ns, not significantly different.

To identify amino acids between hERG1a residues 200 and 228 required to increase hERG1b, we used alanine mutagenesis. We generated five mutants each with five consecutive alanine substitutions spanning the region from residues 200 to 228 in the background of hERG1a N1–228, for example, hERG1a N1–228 (^201^TPAAP^205^ to ^201^AAAAA^205^) (Fig. 8*A*). We coexpressed each of these in *trans* with hERG1b and performed two-electrode voltage-clamp recordings (Fig. 8*B*). We found that each of the five alanine mutants regulated hERG1b deactivation kinetics (Fig. 8*C*) and left shifted the hERG1b G–V curve (Fig. 8*D*). Of these, four of the hERG1a N1–228 mutants (^201^TPAAP^205^ to ^201^AAAAA^205^, ^206^SSESL^210^ to ^206^AAAAA^210^, ^211^ALDEV^215^ to ^211^AAAAA^215^, and ^221^HVAGL^226^ to ^221^AAAAA^226^) increased the hERG1b current amplitude (Fig. 8, *B*, *E* and *F*). In contrast, the hERG1a N1–228 mutant ^216^TAMDN^220^ to ^216^AAAAA^220^ did not measurably increase hERG1b current (Fig. 8, *B*, *E* and *F* and Table 1). Similarly, in whole-cell patch-clamp recordings from HEK293 cells, we found that, compared with hERG1b (Fig. S3*A*), hERG1a N1–228 ^216^TAMDN^220^ to ^216^AAAAA^220^ in *trans* with hERG1b (Fig. S3*B*) had slowed deactivation (Fig. S3*C*), had little measurable effect on the G–V relationship (Fig. S3*D*), and did not measurably increase hERG1b outward currents (Fig. S3, *E* and *F*).

**Figure 9:**
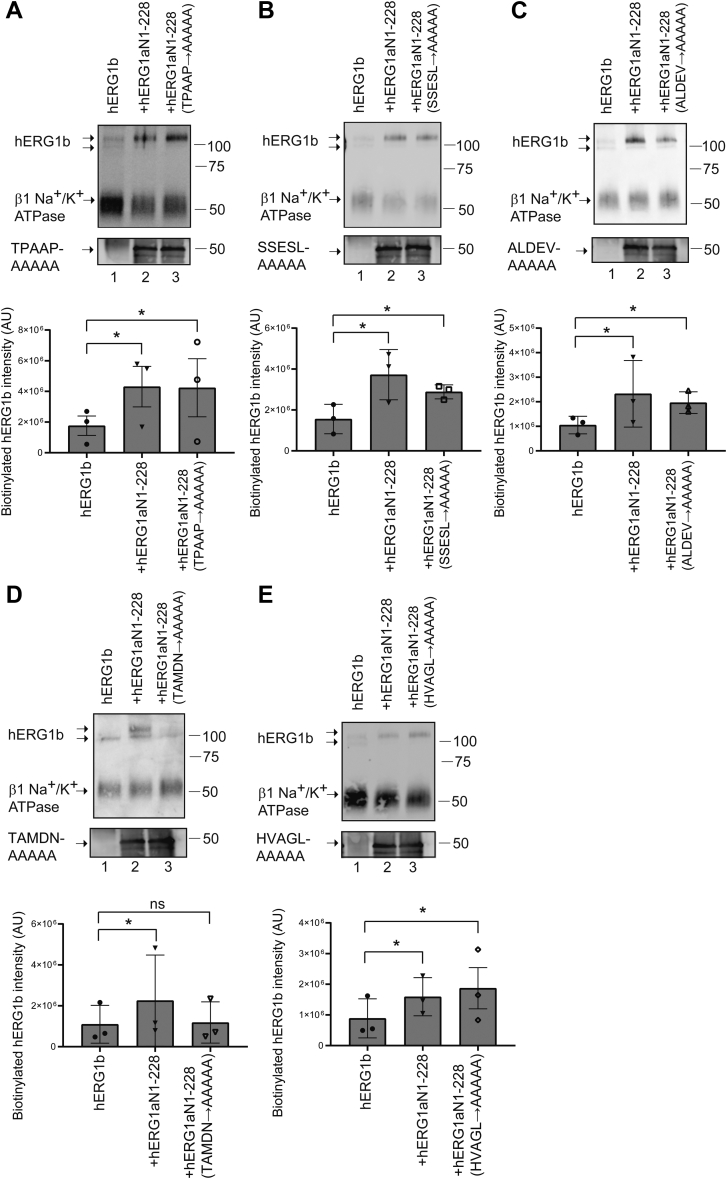
**Alanine mutagenesis revealed amino acids 216–220 of the hERG1a N-linker region were necessary for increased hERG1b maturation and cell surface expression.***A*–*E*, Western blot of surface biotinylated hERG1b coexpressed in *trans* with control vector (lane 1), hERG1a N1–228 (lane 2), or a one of the five hERG1a N1–228s bearing five alanine mutants as labeled (lane 3). hERG1b was detected with anti-hERG KA. The beta subunit of the Na^+^/K^+^ ATPase was detected with an antibody and served as the loading control. An anti-GFP antibody was used to detect hERG1a N1–228 and each N1–228 five alanine mutant as an input control in parallel blots. Plot of densitometry (A.U.) of data from Western blots. N = 3 for each. Error bars represent mean ± SD. ∗ denotes *p* ≤ 0.05 by ANOVA. hERG1, human Ether-á-go-go-Related Gene 1; ns, not significantly different.

We performed biotinylation experiments with each of the hERG1a N1–228 alanine mutants coexpressed in *trans* with hERG1b in side-by-side experiments with hERG1b as a negative control and hERG1a N1–228 in *trans* with hERG1b as a positive control. We found that four of the hERG1a N1–228 mutants (^201^TPAAP^205^ to ^201^AAAAA^205^, ^206^SSESL^210^ to ^206^AAAAA^210^, ^211^ALDEV^215^ to ^211^AAAAA^215^, and ^221^HVAGL^226^ to ^221^AAAAA^226^) increased biotinylated hERG1b at the plasma membrane (Fig. 9,*A*–*C* and *E*). Whereas, in contrast, ^216^TAMDN^220^ to ^216^AAAAA^220^ did not measurably increase biotinylated hERG1b at the membrane (Fig. 9*D*). In summary, the hERG1a N1–228 ^216^TAMDN^220^ to ^216^AAAAA^220^ mutant neither measurably upregulated the magnitude of hERG1b currents (Fig. 8, *B*, *E* and *F*) nor biotinylated hERG1b at the plasma membrane (Fig. 9*D*).

We conclude from these data that the residues ^216^TAMDN^220^ within the hERG1a N-linker in the N-terminal region were required for the increase in hERG1b current. These findings represent a new role for the hERG1a N-linker in upregulating hERG1b.

## Discussion

**Figure 10:**
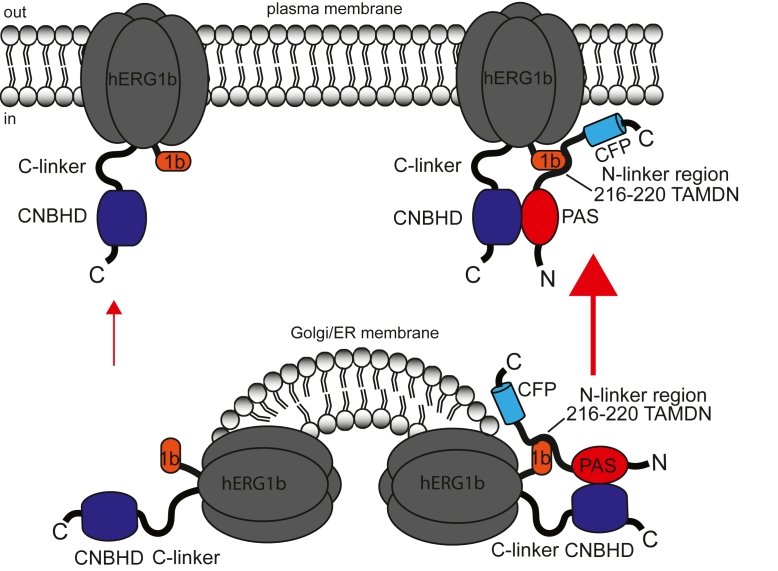
**Scheme of mechanism for hERG1b upregulation at the plasma membrane by hERG1a N-terminal regions.** hERG1b channels are retained internally and only weakly expressed at the plasma membrane (*small arrow*) accounting for small hERG1b currents and weak biotinylated hERG1b density on a Western blot. We propose that the hERG1a N-terminals (*e.g.*, hERG1a N1–228), which encodes the PAS domain (amino acids 1–135) and a portion of the N-linker up to amino acid 228, interacts directly with hERG1b and increases (*large arrow*) hERG1b channels at the plasma membrane, accounting for the larger hERG1b current and robust biotinylated hERG1b density on a Western blot under these conditions. We propose that the hERG1a N1–228 interaction with hERG1b involves a direct interaction of the hERG1a PAS (1–135) domain with the CNBHD of hERG1b, which regulates and slows the deactivation time course and that a part of the hERG1a N-linker, where amino acids 216 to 200 are necessary, interacts with the hERG1b N-terminal “1b domain” to upregulate hERG1b at the plasma membrane. CNBHD, cyclic nucleotide–binding homology domain; hERG1, human Ether-á-go-go-Related Gene 1; PAS, Per–Arnt–Sim.

To explain our data, we propose that the hERG1a N-terminal PAS domain–N-linker region increases hERG1b current by interacting with and stabilizing (*large arrow*) hERG1b channels at the plasma membrane (Fig. 10). In contrast, hERG1b alone only weakly makes homomeric channels at the plasma membrane (*small arrow*) and instead is preferentially retained at intracellular membranes (this study and Refs. (19, 20)). We propose that the hERG1a PAS domain–N-linker region stabilizes hERG1b through a mechanism where the PAS domain of hERG1a interacts directly with the C-linker and CNBHD of hERG1b (this study and Ref. (17)), and the N-linker region of hERG1a interacts with the N-terminal domain of hERG1b (Fig. 10). We propose that residues ^216^TAMDN^220^ in the hERG1a N-linker are required for the functional upregulation of hERG1b, perhaps by being necessary for the interaction of the N-linker with hERG1b.

In heteromeric channels comprising full-length hERG1a and hERG1b subunits, such as those in the heart (1, 2), we propose that the intracellular N-terminal domains are arranged such that the PAS domain of hERG1a makes an intersubunit interaction with the CNBHD of hERG1b and that the hERG1a N-linker domain interacts with the hERG1b N-terminal domain. Since hERG1b expression at the plasma membrane is enhanced by hERG1a N1–354 and hERG1a N1–228, we suspect that the interaction occurs during biogenesis and folding, and this is supported by previous work indicating that the hERG1a N terminus and hERG1b N terminus associate cotranslationally (19). However, we cannot rule out the alternative possibility that hERG1a N1–354 or hERG1a N1–228 only associate with hERG1b at the plasma membrane. We speculate that an interaction of the hERG1a N-linker region centered on residues ^216^TAMDN^220^ with the hERG1b “1b domain” helps to promote the surface expression of hERG1b in the presence of wildtype hERG1a subunits. This interaction is not present in homomeric hERG1a channels, and thus, the N-linker region might not be in its native form in homomeric hERG1a channels, which may explain why the N-linker needed to be deleted (15) in order to obtain well-behaving hERG1a protein for cryo-EM studies. Likewise, perhaps the N-terminal “1b domain” of hERG1b is not in a native conformation in homomeric hERG1b channels.

Our results here fit well with previous conclusions about hERG1a and hERG1b N-terminal domain interactions and the location of a region in hERG1a that is necessary for upregulation of hERG1b. In a previous study, a biochemical interaction between fusion proteins encoding the hERG1a N-terminal domain–S2 transmembrane domain and hERG1b N-terminal domain–S2 transmembrane domain was detected (19). Here, we refine the identity of the interacting regions by showing that the hERG1a N-terminal region including the PAS domain and most of the N-linker (hERG1a N1–354) and the hERG1b N-terminal domain (hERG1b N1–56) were sufficient to form an interaction (Figs. 4 and 5). In a previous study, full-length hERG1a and hERG1a subunits with a deletion of amino acids 2 to 200 both increased the maturation of hERG1b, indicating that an enhancer region for hERG1b was upstream of residue 200 (20). In this study, we mapped the region necessary for hERG1a to upregulate hERG1b to amino acids ^216^TAMDN^220^ in the hERG1a N-linker region (Figure 6, Figure 7, Figure 8, Figure 9).

Diarginine motifs in the hERG1b unique N-terminal region are important for endoplasmic reticulum retention of hERG1b and are masked by hERG1a, leading to an increase in hERG1b at the plasma membrane (20), which we confirm here (Fig. S4). We tested whether the hERG1b diarginine motif located at amino acids 15 to 17 (*i.e.*, ^15^RPR^17^) were required for the increase in hERG1b by the hERG1a N-terminal region. We report that a hERG1a N-terminal region (hERG1a N1–228) increased wildtype hERG1b (*i.e.*, ^15^RPR^17^) as well as hERG1b bearing diarginine motif mutants at amino acids 15 and 17 (*i.e.*, ^15^NPN^17^, ^15^DPD^17^, or ^15^KPK^17^) at the plasma membrane as measured with biotinylation (Fig. S4*A*). Likewise, hERG1b ^15^AAA^17^ channels, in which all residues in the RPR motif were mutated to three alanine residues, were increased by hERG1a N1–228, but not hERG1a N1–135 (Fig. S4*B*), suggesting that the hERG1a N1–228–dependent increase in hERG1b is by a mechanism independent of hERG1b diarginine motifs and remains specific to the regions between residues 136 and 228. We speculate that another part of hERG1a, excluding hERG1a N-terminal residues 1 to 228, masks hERG1b diarginine motifs.

The direct interaction of mammalian ERG1a and ERG1b is crucial for cardiac physiology. Mammalian ERG1a and ERG1b subunits interact to form native cardiac I_Kr_ in the mammalian heart, including human heart, and manipulation of I_Kr_ to favor hERG1b-like kinetics or hERG1a-like kinetics is proarrhythmic (1, 2, 3, 8, 19, 20, 21, 22, 23). Our results indicate that the N-linker region of hERG1a is a new player in this assembly mechanism, and thus, disruption of the N-linker mechanism may be a target for inherited LQTS mutations and a target for therapeutic interventions.

## Experimental procedures

### Molecular biology

The hERG1a and hERG1b constructs used here were described previously (12, 17, 24). hERG1b and hERG1bΔ2–36 and hERG1b 15-AAA-17 were fused to Citrine fluorescent protein and all hERG1a N-terminals were fused to monomeric CFP (mCFP). Some recombinant hERG1a N-terminal regions were described previously (17, 24). The hERG1a N1–200-mCFP, hERG1a N1–228-mCFP and hERG1a N1–228mCFP ^201^TPAAP^205^ to ^201^AAAAA^205^, ^206^SSESL^210^ to ^206^AAAAA^210^, ^211^ALDEV^215^ to ^211^AAAAA^215^, ^216^TAMDN^220^, ^221^HVAGL^226^ to ^221^AAAAA^226^, and hERG GST fusion protein plasmids and 6xHis/FLAG-tagged fusion protein plasmids were made with customized primers and generated commercially (BioInnovatise, Inc). The mCFPs were included to aid in visual detection, detection on Western blots, and as FRET donors. Constructs used in FRET 2-hybrid studies were either previously described (12) or generated commercially (*i.e.*, hERG1b N1–56-Citrine and hERG1 C666–1159-Citrine) (BioInnovatise). hERG1b, hERG1b 15-NPN, hERG1b 15-DPD, and hERG1b 15-KPK used in Fig. S4 were not fused to a fluorescent protein and were the kind gift of Dr G.A. Robertson (20). For oocyte expression, all constructs were in the pGH19 vector, and for HEK293 cell expression, all constructs were in the pcDNA3.1 vector. RNA for oocyte injection was transcribed *in vitro* with the T7 mMESSAGE mMACHINE kit (Invitrogen).

### Electrophysiology

Two-electrode voltage-clamp and whole-cell patch-clamp studies were performed as previously described (24, 27). Oocytes were obtained from a commercial source (Ecocyte, Inc) and injected with 49 nl of RNA and incubated for 24 to 72 h in ND-96 buffer + 50 μg/ml gentamycin prior to electrophysiology. A ratio of 2:1 for N-terminal domains to channel RNA was used in coexpression studies. TEVC was performed using an oocyte clamp (OC-725C; Warner Inst) and an A/D converter (ITC-18; InstruTech). Recording electrodes were fashioned with a micropipette puller (Sutter), filled with 3M KCl, and had resistances of 0.5 to 1.2 MΩ. The external (bath) solution was (millimolar) 4 KCl, 94 NaCl, 1 MgCl_2_, 0.3 CaCl_2_, and 5 Hepes with pH 7.4.

HEK293 cells were transiently transfected with TransIT-LT1 reagent (Mirus) with hERG1a N-terminal region cDNAs in a 2:1 ratio with hERG1b cDNAs in coexpression experiments. Whole-cell recordings were performed 24 to 48 h after transfection using a patch-clamp amplifier with a built-in A/D converter (EPC10; HEKA, Inc). Microelectrodes had resistances of 2 to 3 MΩ after filling with pipette (internal) solution of (in millimolar) 130 KCl, 1 MgCl_2_, 5 EGTA, 5 MgATP, and 10 Hepes, pH 7.2. The bath (external) solution was (in millimolar) 137 NaCl, 4 KCl, 1.8 CaCl_2_, 1 MgCl_2_, 10 glucose, 10 Hepes, pH 7.4. Capacitance was compensated on-line. No leak subtraction was performed.

Electrophysiological recordings were made at room temperature and acquired with Patchmaster Data Acquisition software (HEKA Electronic). Currents were analyzed offline using Igor Pro (WaveMetrics). G–V data were fit with a Boltzmann function (y = 1/[1 + e[(V_1/2_ − V)/k]]) in which V_1/2_ is the half-maximum potential for activation and k is the slope factor. Deactivating currents were fit with a single exponential function. (y = Ae[–t/tau]) where t is time and tau is the time constant of deactivation. All data are presented as the mean ± SD, and n is the number of cells.

Unless otherwise noted, one-way ANOVA with Tukey’s test was performed to determine statistical significance using GraphPad Prism (Dotmatics). A value of *p* < 0.05 was considered statistically significant.

### Biochemistry and biotinylation

Biochemical detection of hERG1a and hERG1b proteins was performed using biotinylation as previously described (24). Briefly, HEK293 cells were transfected with hERG1b or hERG1a cDNAs using TransIT-LT1 transfection reagent. After 24 to 48 h, cells were incubated with extracellular application of EZ-link Sulfo-NHS-SS-biotin (1 mg/ml; Pierce), and proteins were purified with biotin–streptavidin beads. Bead-bound proteins were separated by SDS-PAGE, transferred to nitrocellulose, and immunoblotted with a primary hERG antibody (anti-hERG KA; ENZO) and a horseradish peroxidase–conjugated secondary antibody (goat anti-rabbit; Life Technologies). An antibody was used to detect the beta subunit of the endogenous Na/K ATPase (MA3-930; Invitrogen), which was used as a loading control for biotinylated blots (*e.g.*, Fig. 2*G*). Blots were visualized with a gel imager (BioRad XRS) using chemiluminescence (SuperSignal West Femto; Thermo Fisher Scientific). Anti-GFP (anti-GFP ab290; Abcam) probed blots were run in parallel to detect hERG1a N-terminal inputs (*e.g.*, Fig. 2*G*). In some experiments, samples without the biotin purification step were run in parallel and blotted with an antibody to protein disulphide isomerase (anti–protein disulphide isomerase; ab3672; Abcam) as a loading control (*e.g.*, Fig. S4*A*). All data are presented as the mean ± SD, and n is the number of cells. One-way ANOVA with Tukey’s test was performed to determine statistical significance. A value of *p* < 0.05 was considered statistically significant.

### Biochemical pull-down interaction assay

Biochemical interaction assays were performed as previously described (13). Briefly, GST hERG1a N-terminal domains or GST-only negative control constructs were grown in BL21(DE3)-competent cells (Agilent Technologies) until they reached exponential growth. Protein expression was induced using IPTG (0.4 mM; Research Products International) with shaking at 30 °C overnight (O.N.). Bacteria were harvested with centrifugation, resuspended in buffer S (50 mM Tris, pH 8, 150 mM NaCl, 25 mM imidazole, 0.5% CHAPS, and 0.25% Tween-20; Sigma–Aldrich) and lysed with a sonicator. The products were centrifuged at high speed, and the supernatants were applied to glutathione beads (GE Healthcare), washed with buffer S, and the protein concentration was measured using a Bradford assay.

The 6xHis fusion proteins were grown in M15 cells (VWR) and prepared as aforementioned except that after IPTG induction, proteins were grown O.N. at 18 °C. Materials were cleared by a high-speed spin as aforementioned and applied to a nickel column (HiTrap Chelating HP; GE Healthcare), washed, and eluted in buffer S with imidazole (500 mM).

To perform interaction assays, the bead-bound GST fusion proteins and 6xHis fusion proteins were combined in buffer S with 0.5% CHAPS detergent. Proteins were incubated O.N. at 4 °C on a rotating mixer. Samples were washed with buffer S, and proteins were stripped from beads by incubation in gel loading buffer with beta-mercaptoethanol and loaded to 4 to 15% gels (Criterion; Bio-Rad) for SDS-PAGE. GST inputs were assessed by Coomassie blue staining of gels. Interacting proteins were resolved with SDS-PAGE, transferred to nitrocellulose, and blotted with an anti-FLAG antibody (Sigma–Aldrich) and HRP-linked secondary antibody (Themo Fisher Scientific), and visualized with a Chemiluminescent Detection Kit (SuperSignal West Femto; Thermo Fisher Scientific) and a gel-imaging system (ChemiDoc XRS; Bio-Rad).

### FRET two-hybrid assay

We used a FRET hybridization assay to test for associations between two isolated ion channel domains (or regions), where one domain was fused to mCFP as a FRET donor, and a second domain was fused to monomeric Citrine as a FRET acceptor (12, 24). Then, the two separate cDNAs (*e.g.*, hERG1a N1–354-mCFP and hERG1b N1–56-Citrine, as depicted in Fig. 5*A*), were transfected into HEK293 cells. After 24 to 48 h, fluorescence images were measured from cells using an inverted microscope (Nikon TE-2000) with a 120 W lamp (X-Cite 120) as the light source, a 60× objective with numerical aperture 1.45 (Nikon), a custom “FRET” cube with excitation filter, dichroic and emission filter D436/20, 455dclp and D460lp, respectively (made from a CFP cube modified with a long-pass emission filter to also detect Citrine emission), and a YFP cube (Chroma Technology Corp) with HQ500/20, Q515lp, and HQ520lp. Emission was detected with a spectragraph (SpectraPro 2150i; Acton Research) and a charge-coupled device camera (Roper 512B; Roper Scientific) connected in-line to the microscope emission port. MetaMorph 6.3r7 (Universal Imaging/Molecular Devices) and Monochromator Control (Acton) acquisition and analysis software packages were used to record fluorescence images and spectra. All data are presented as the mean ± SD, and n is the number of cells. One-way ANOVA with Tukey’s test was performed to determine statistical significance. A value of *p* < 0.05 was considered statistically significant.

## Data availability

Exemplar current recordings, fluorescence spectroscopy traces, and Western blot images are all located within the article. All data are contained in plots within the article. Any additional raw data used to generate plots can be requested from Dr Matt Trudeau (mtrudeau@som.umaryland.edu).

## Supporting information

This article contains supporting information.

## Conflict of interest

The authors declare that they have no conflicts of interest with the contents of this article.

## Supporting information

Supplemental Figures

FigS1

FigS2

FigS3

FigS4
