# Supplementary material for: The N-linker region of hERG1a upregulates hERG1b potassium channels
Source: J Biol Chem. 2022 Jul 5;298(9):102233. doi: 10.1016/j.jbc.2022.102233 (PMC9428852; doi:10.1016/j.jbc.2022.102233)
Supplement: Supplemental Figures [file mmc1.docx]

**Figure S1. The hERG1a N-terminal PAS domain (hERG1a N1-135) regulated deactivation gating but did not increase outward hERG1b currents in HEK293 cells.** A) Whole-cell patch-clamp recordings of hERG1b (closed circles) and B) hERG1a N1-135 co-expressed *in trans* with hERG1b (closed squares) using the voltage protocol indicated. Scale bar is 100 nA and 1 second. C) Time constant of deactivation from fit to tail currents at -50mV. **** denotes p<.0001 by Student’s t-test. D) Conductance-voltage (G-V) plot. E) Current-voltage (I-V) plot. F) Histogram of peak current amplitude at depolarizing voltage. N ≥ 3 for each. Error bars are mean ± SD. ns denotes not significantly different.

**Figure S2**. **A** **hERG1a N-terminal composed of amino acids 1-228 (hERG1a N1-228) increased hERG1b currents in HEK293 cells.** A) Whole-cell patch-clamp recordings of hERG1b (closed circle) and B) hERG1a N1-228 co-expressed *in trans* with hERG1b (closed triangle) using the voltage protocol as indicated. Scale bar is 100 nA and 1 second. C) Time constant of deactivation from fit to tail currents at -50mV. D) Conductance-voltage (G-V) plot. E) Current-voltage (I-V) plot. F) Histogram of peak current amplitude at depolarizing voltage. N ≥ 3 for each. Error bars are mean ± SD. * denotes p<.05 by Student’s t-test.

**Figure S3.** **Alanine mutagenesis revealed amino acids 216-220 of the hERG1a N -linker were necessary for increased hERG1b current amplitude in HEK293 cells.** Whole-cell patch-clamp recordings of A) hERG1b (closed circle) and B) hERG1a N1-228 ^216^TAMDN^220^  to ^216^AAAAA^220^ co-expressed *in trans* with hERG1b (open triangle) C) Time constant of deactivation from fit to tail currents at -50mV. * denotes p<.05 by Student’s t-test. D) Conductance-voltage (G-V) plot. E) Current-voltage (I-V) plot. F) Histogram of peak current amplitude at depolarizing voltage. N ≥ 3 for each. Error bars are mean ± SD. ns denotes not significantly different.

**Figure S4. hERG1a N-terminal regions (hERG1a N1-228) enhanced surface expression of hERG1b channels with mutations in the di-arginine (RXR) ER retention motif.** A) Western blot of biotinylated hERG1b proteins as indicated, hERG1b 15-RPR (wild-type hERG1b with RXR motif at amino acids 15-17), hERG1b 15-NPN, hERG1b 15-DPD and hERG1b 15-KPK expressed alone or co-expressed *in trans* (+) with hERG1a N1-228. The loading control was PDI. In this experiment, hERG1b and hERG1b RXR mutants (a kind gift from Dr. G.A. Robertson) were not labelled with Citrine. B) Western blot of biotinylated hERG1b-Citrine with 3 alanine mutations at the RPR motif (RPR to AAA at amino acids 15-17) expressed with either empty vector control, the hERG1a PAS domain (hERG1a N1-135) or the hERG1a PAS domain with a part of the N-linker region (hERG1a N1-228) as denoted with + symbols. Loading control was the beta subunit of the Na/K ATPase. N=3.
