# Supplementary figures and images for: The N-linker region of hERG1a upregulates hERG1b potassium channels
Source: J Biol Chem. 2022 Jul 5;298(9):102233. doi: 10.1016/j.jbc.2022.102233 (PMC9428852; doi:10.1016/j.jbc.2022.102233)

Figure S1

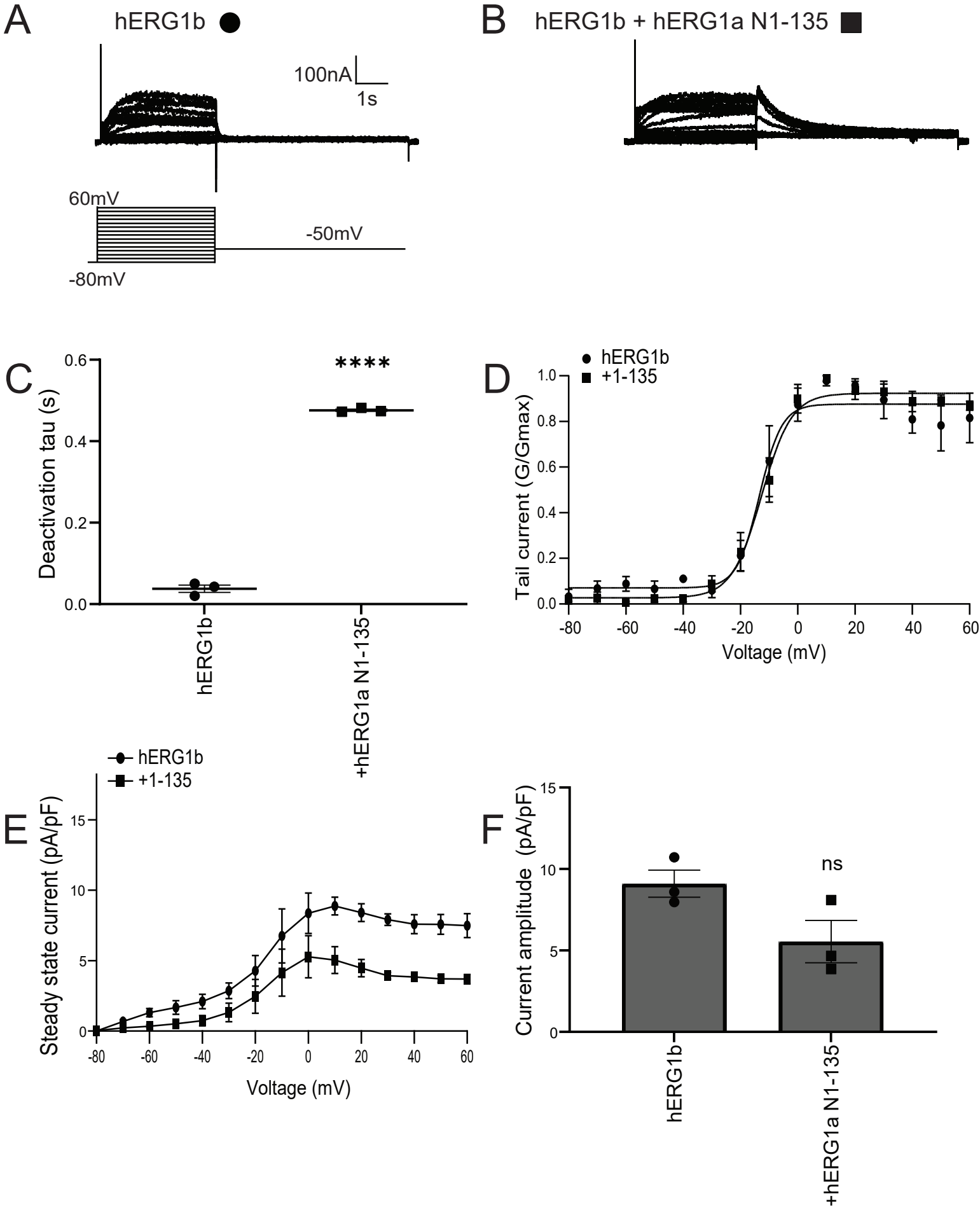

Supplement: FigS1 [file mmc2.pdf]

A

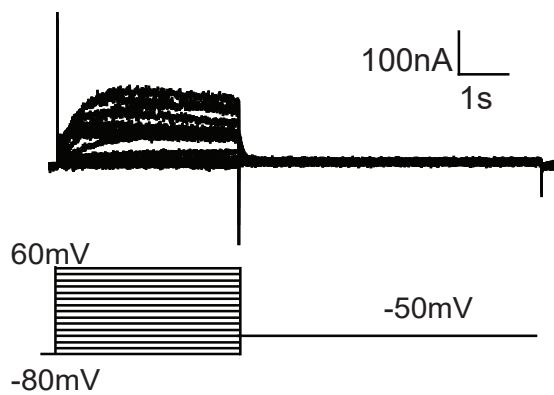

B

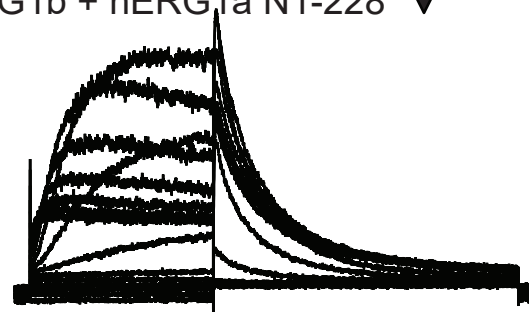

C

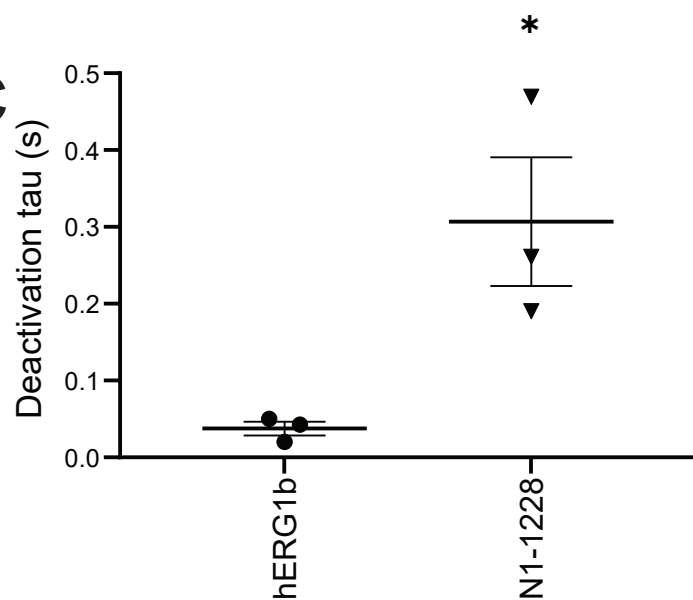

D

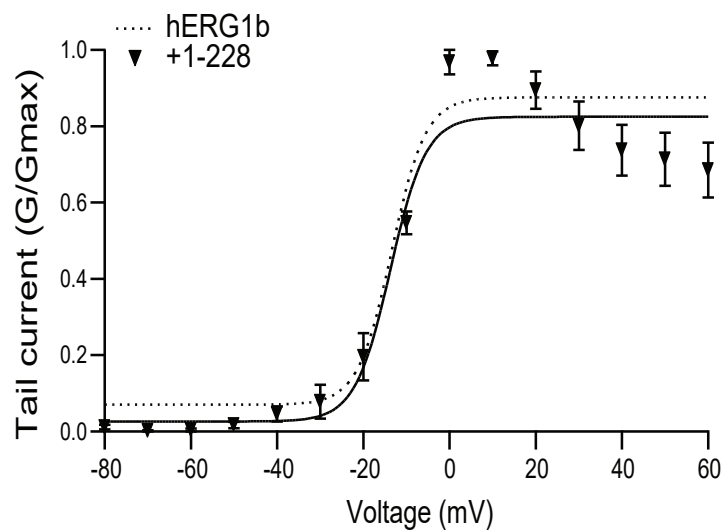

E

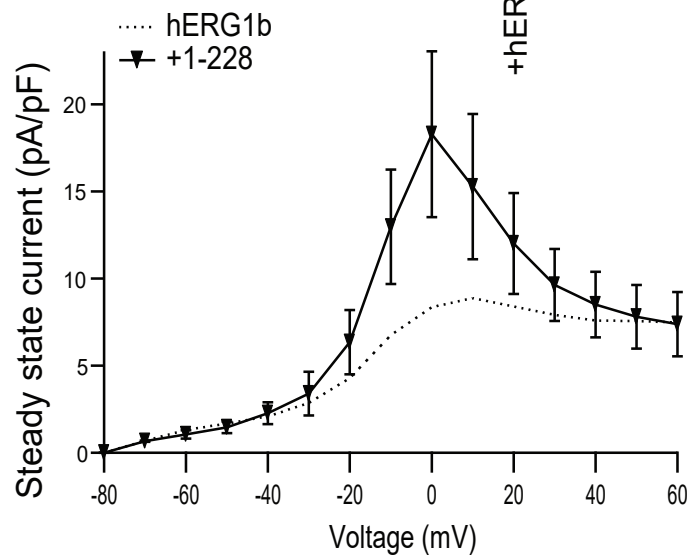

F

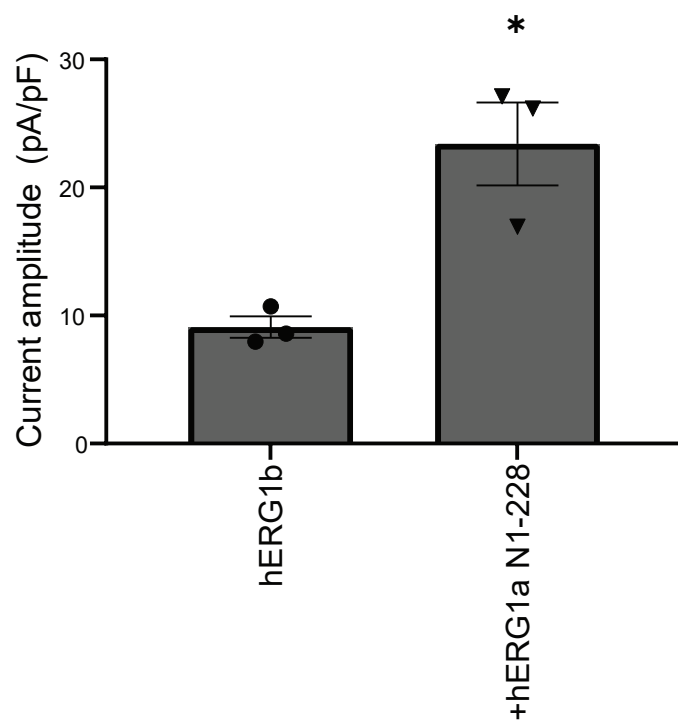

Supplement: FigS2 [file mmc3.pdf]

Figure S3

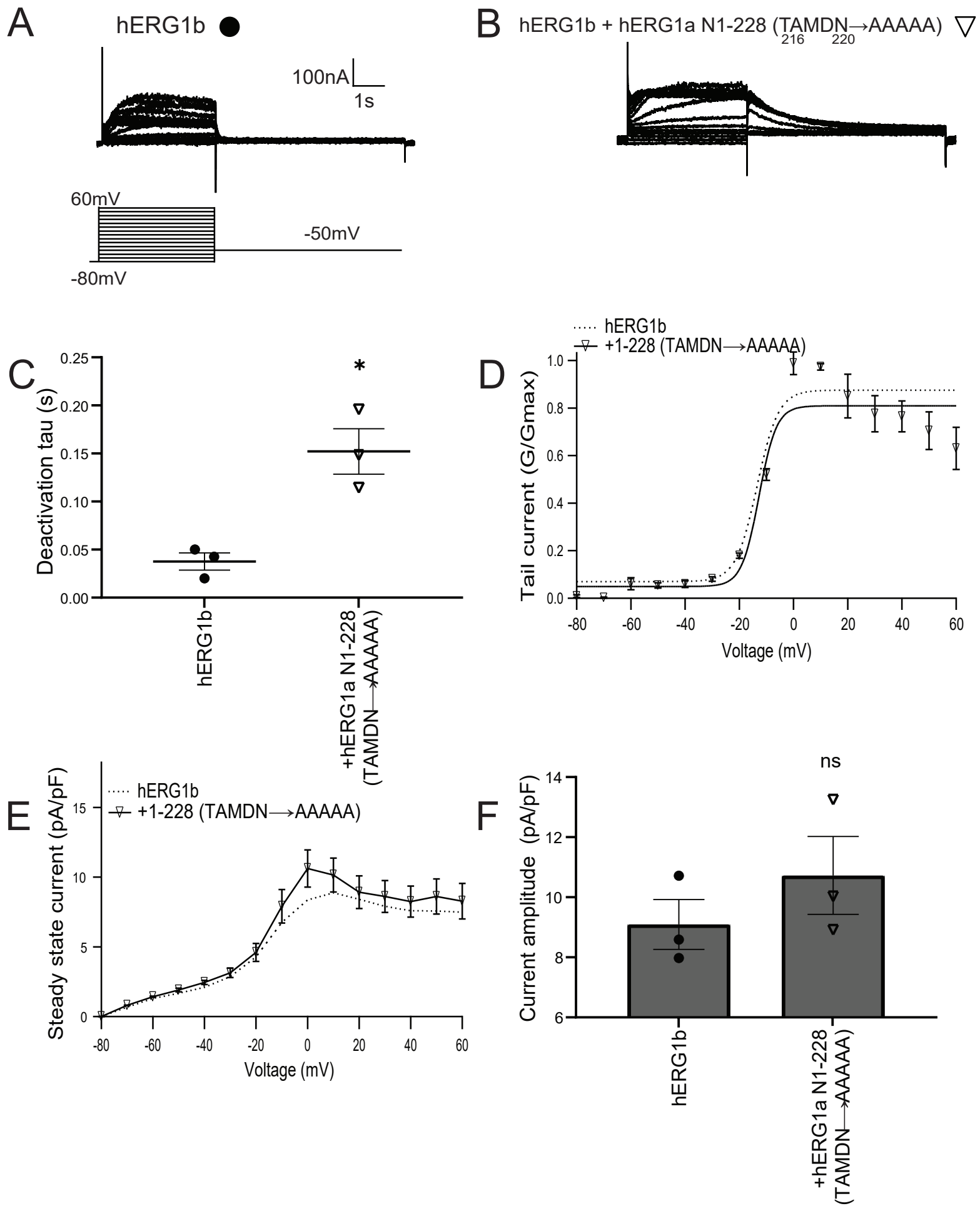

Supplement: FigS3 [file mmc4.pdf]

Figure S4

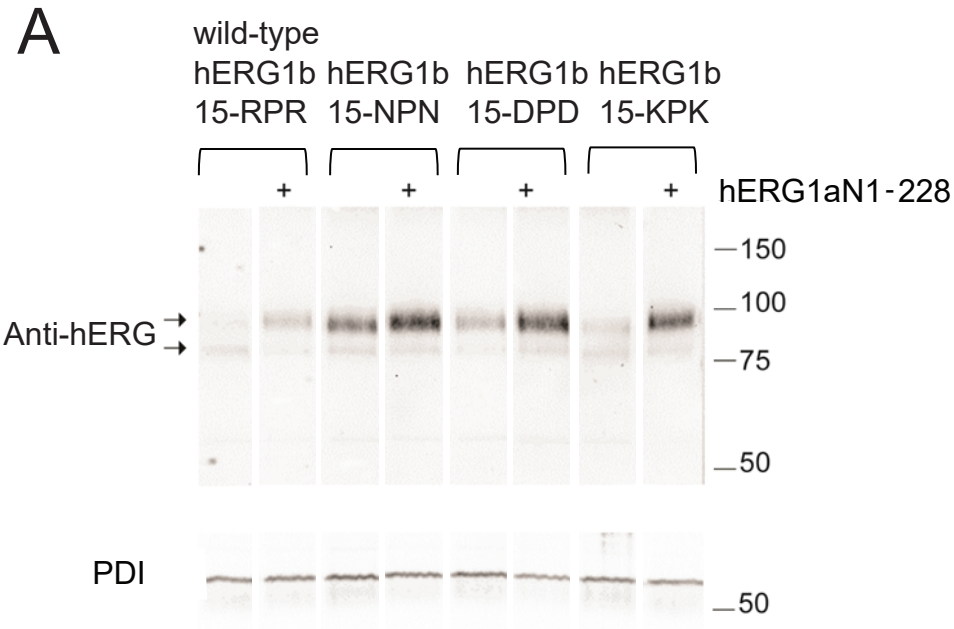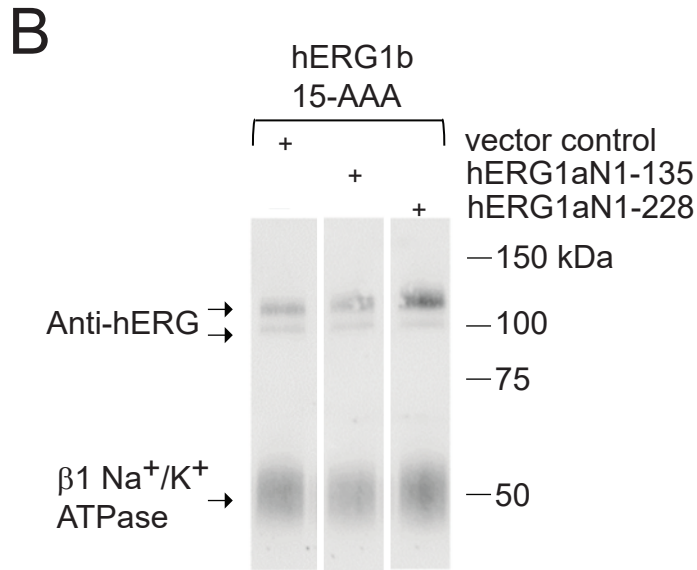

Supplement: FigS4 [file mmc5.pdf]
